# Two chromosome-level genomes of *Smittia aterrima* and *Smittia pratorum* (Diptera, Chironomidae)

**DOI:** 10.1038/s41597-024-03010-y

**Published:** 2024-02-03

**Authors:** Yue Fu, Xiangliang Fang, Yunli Xiao, Bin Mao, Zigang Xu, Mi Shen, Xinhua Wang

**Affiliations:** 1 Hubei Key Laboratory of Economic Forest Germplasm Improvement and Resources Comprehensive Utilization, Hubei Collaborative Innovation Center for the Characteristic Resources Exploitation of Dabie Mountains, Hubei Zhongke Research Institute of Industrial Technology, College of Biology and Agricultural Resources, Huanggang Normal University, Huanggang City, Hubei 438000 China; 2 College of Life Sciences, Nankai University, Tianjin, 300071 China

**Keywords:** Entomology, Biodiversity

## Abstract

Chironomids are one of the most abundant aquatic insects and are widely distributed in various biological communities. However, the lack of high-quality genomes has hindered our ability to study the evolution and ecology of this group. Here, we used Nanopore long reads and Hi-C data to produce two chromosome-level genomes from mixed genomic data. The genomes of *Smittia aterrima* (SateA) and *Smittia pratorum* (SateB) were assembled into three chromosomes, with sizes of 78.45 Mb and 71.56 Mb, scaffold N50 lengths of 25.73 and 23.53 Mb, and BUSCO completeness of 98.5% and 97.8% (n = 1,367), 5.68 Mb (7.24%) and 1.94 Mb (2.72%) of repetitive elements, and predicted 12,330 (97.70% BUSCO completeness) and 11,250 (97.40%) protein-coding genes, respectively. These high-quality genomes will serve as valuable resources for comprehending the evolution and environmental adaptation of chironomids.

## Background & Summary

The non-biting chironomid midges (Diptera: Chironomidae) have developed unique adaptations enabling them to endure and prosper in severe abiotic conditions, such as extreme temperatures, oxygen deprivation, pollution, high salinity, and varying pH levels, as well as complete desiccation (Henriques-Oliveira *et al*., 2003; Osbourne *et al*., 2000; Vos *et al*., 2002; Pinder, 1986). As remarkably resilient aquatic organisms, research has indicated that chironomid survival in diverse water habitats necessitates specific detoxification enzymes^1–4^.

To gain a better understanding of the fundamental mechanisms of tolerance, researchers have increasingly focused on the genomes of organisms capable of thriving in extreme environments. Three notable examples include the chironomid midges *Polypedilum vanderplanki*, which can survive near complete water loss in its larval form, *Belgica antarctica*, which exhibits remarkable freeze tolerance, and *Propsilocerus akamusi*, a species recognized for its ability to tolerate pollution^5–7^. Such genomes had shaped by their environment and the adaptations they undergo to survive and reproduce. The Orthocladiinae, which is the largest subfamily within Chironomidae, is characterized by a remarkable diversity of species that have evolved varied ecological and physiological adaptations to their respective environments. Among the Orthocladiinae, the genus *Smittia*, encompassing species such as *S. aterrima* and *S. pratorum*, was notable for its thriving in littoral environments owing to their unique terrestrial/amphibious way of life, as the majority of chironomid larvae are aquatic^8–10^. Besides, research on *Smittia* sp. has been instrumental in advancing our understanding of parthenogenesis, polytene chromosomes, embryonic development, and nucleolar RNA synthesis, demonstrating significant scientific importance^11–16^.

With the multiple purposes of generating genomic resources to investigate chironomid genome evolution, chromosomal composition, and tolerant specialization, we utilized Oxford Nanopore Technologies (ONT) and high-throughput chromosome conformation capture (Hi-C) sequencing to produce two chromosome-level reference genomes for *S. aterrima* and *S. pratorum*. We performed genome assembly, and annotation, and conducted evolutionary analyses. Our work generates a valuable chromosome-level genomic resource for chironomids, establishing a foundation for future research into the environmental tolerance mechanisms of these insects.

## Methods

### Sample collection and sequencing

Live male adult specimens of *S. aterrima* and *S. pratorum* were collected at Baitan Lake (Huanggang City, Hubei Province, China, 30.463250°N, 114.942184°E). After sample collection, the whole bodies of samples were immediately immersed into liquid nitrogen and stored at −80 °C. There were 300 male adults (about 200 male adults of *S. aterrima* and 100 male adults of *S. pratorum* were mixed together) used for genome sequencing.

**Fig. 1:**
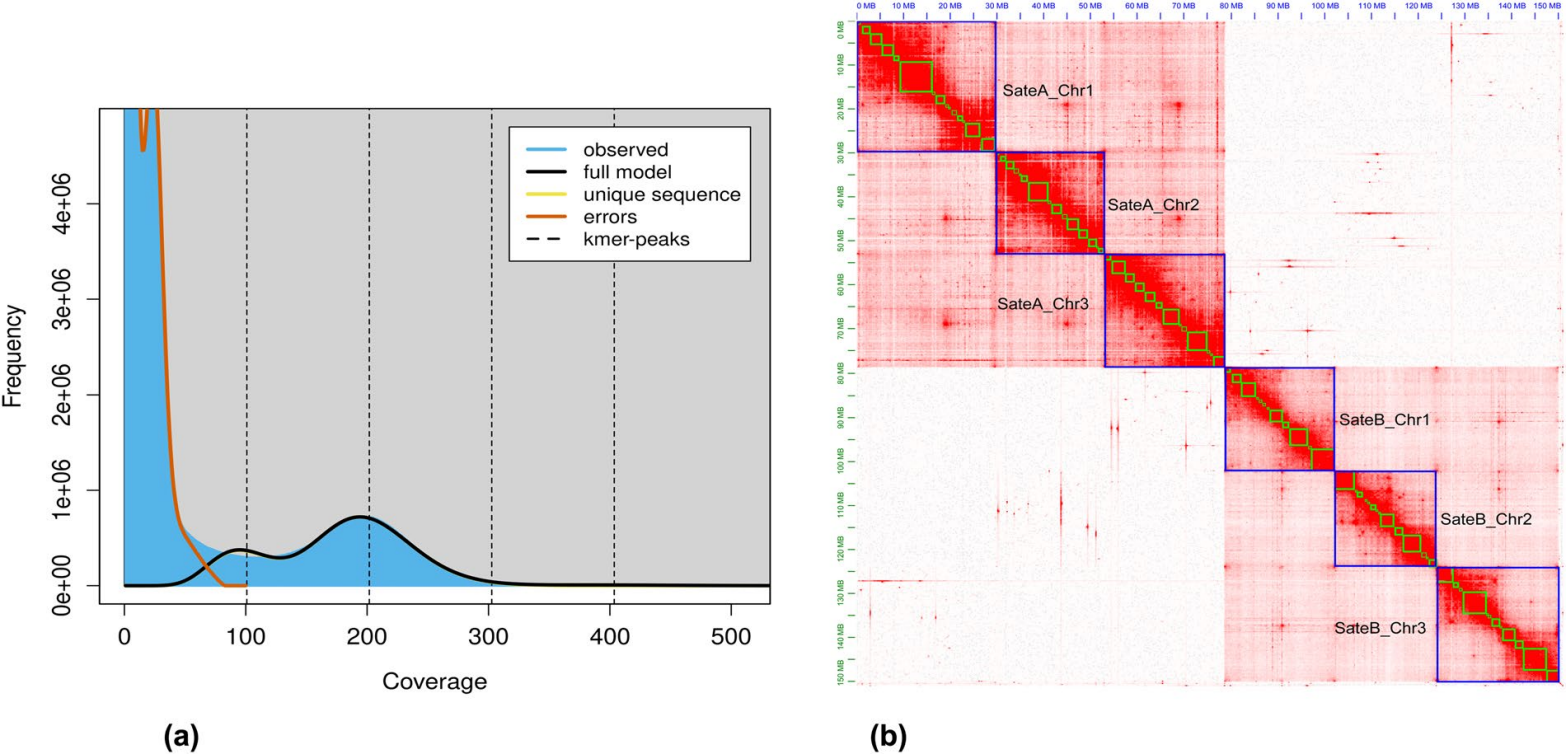
(**a**) The survey results were obtained based on the 21 k-mer analysis. (**b**) Heatmap showing genome-wide all-by-all Hi‐C interactions (three chromosomes for each species of *S. aterrima* (SateA) and *S. pratorum* (SateB)). The map indicates that intrachromosomal interactions (red blocks in the diagonal) were stronger than interchromosomal interactions.

DNA was extracted using the 1D DNA Ligation Sequencing kit SQK-LSK109. RNA was extracted with the TRIzol™ Reagent kit. After the determination of the DNA quality and quantity, a paired-end sequencing library (350 bp in length) was constructed and sequenced using the Beijing Genomics Institute (BGI), and the library construction was completed by Berry Genomic Corporation (Beijing, China). In addition, a Single Molecule Real-Time DNA library was prepared for sequencing using SQK-LSK109 Kit with an insert size of 30 kb. The Oxford Nanopore third-generation sequencing was completed by BenaGen Corporation in Wuhan, China. The RNA library was constructed with Illumina TruSeq RNA v2 Kit according to the manufacturer’s instructions, and the three-generation full-length (ONT) RNA was extracted by (DP441) RNA prep Pure Plant Plus Kit to construct an ONT PromethION library, was completed by Berry Genomic Corporation in Beijing, China. Hi-C libraries were constructed according to the improved Hi-C procedures^17^. including cross-linking of formaldehyde, restriction enzyme digestion, ends repair of fragments, DNA cyclization, DNA purification, and other steps with MboI as the restriction enzyme. Finally, we obtained 93.95 Gb of sequencing data, comprising 28.11 Gb of Illumina reads, 21.16 Gb of Nanopore reads, 13.40 Gb of Hi-C data, and 31.28 Gb of RNA data, which consisted of 21.78 Gb of Illumina sequencing and 9.50 Gb of ONT sequencing. The mean/N50 lengths of the Nanopore and ONT reads were 6.01/21.65 kb and 0.99/1.41 kb, respectively (Table 1). The 28.11 Gb Illumina DNA data was retained after the quality control process and then used for the genome survey. The k-mer (k = 21) analysis demonstrated that the genomes with a low heterozygous ranging from 0.70%‐0.85% (Fig. 1a, Table 2), and the estimated size was about 83.52‐84.51 Mb.

**Table 1:** Raw data from the different sequencing.

Sequencing type	Number of sequences	Raw data (bp)	Average length (bp)	N50 length (kb)
Survey (BGI)	187,403,696	28,110,554,400	150	—
RNA (Illumina)	145,173,266	21,775,989,900	150	—
Nanopore	3,518,817	21,164,492,863	6,014.66	21.65
Hi-C	89,348,330	13,402,249,500	150	—
ONT RNA	9,506,429	9,500,855,646	999.41	1.41

Note: ONT, Oxford Nanopore Technologies.

**Table 2:** Results of the Suvery analysis.

property	Min	Max
Homozygous (aa)	99.15%	99.30%
Heterozygous (ab)	0.70%	0.85%
Genome Haploid Length (bp)	83,521,170	84,511,873
Genome Repeat Length (bp)	2,865,598	2,899,589
Genome Unique Length (bp)	80,655,572	81,612,284
Model Fit	92.97%	94.61%
Read Error Rate	1.26%	1.26%

### Genome size estimation and assembly

Quality control of the BGI data was carried out by BBTools v38.82^18^: “clumpify.sh” is used to remove repeats; “bbduk.sh” is used for specific quality control, i. e. removing sites with a base mass score below 20 (>Q20), filtering sequences less than 15 bp, removing poly-A/G/C ends over 10 bp, and correcting bases using the overlap region (overlapping reads). The k-mer frequencies were assessed using “khist.sh” (BBTools) with a length set to 21 k-mer. The k-mer analysis was then performed using GenomeScope v2.0^19^, with a maximum k-mer coverage of 1,000 (“-m 1000”).

For genomic contig assembly, the ONT raw data were error-corrected by NextDenovo v2.5.0 (https://github.com/Nextomics/NextDenovo), filtered for contaminated sequences using Kraken v2.1.2^20^, and then assembled using NextDenovo software with parameters read_cutoff = 1k. Sequences below 1 kb in the raw data were filtered. One round of long sequence correction using Inspector v1.2^21^ and two rounds of short sequence correction with NextPolish v1.3.0^22^ to obtain the corrected genome sequence and further improve the assembly accuracy (Table 3). Minimap2 v.2.17^23^ was employed as the read *Fundamental* mapper during long and short-read polishing stages.

**Table 3:** Result statistics of the genome assembly.

Assembly	Total length (Mb)	Number of scaffolds/contigs	scaffold/contigs N50 length (Mb)	Longest scaffold/contigs (Mb)	GC (%)	BUSCO (n = 1,367) (%)			
						C	D	F	M
NextDenovo	151.48	78/78	3.387/3.387	9.627/9.627	39.16	98.4	56.6	0.4	1.2
NextPolish	151.31	78/78	3.381/3.381	9.617/9.617	39.16	99.1	83.5	0.1	0.8
3D-DNA	151.33	89/207	25.732/2.582	29.787/7.074	39.16	99.2	84.9	0	0.8

Note: BUSCO, Benchmarking Universal Single-Copy Orthologs; C, complete BUSCOs; D, complete and duplicated BUSCOs; F, fragmented BUSCOs; M, missing BUSCOs.

In order to obtain clean data, the adapter sequences of raw reads were trimmed and low-quality reads were removed using Juicer v1.6.2^24^. Subsequently, the clean reads were mapped to the draft genome into the chromosome using 3D-DNA. Juicebox v1.11.08^24^ was used to correct possible errors (such as misjoins, translocations, and inversions) in the candidate assembly by visualizing Hi-C heatmaps. Judging from the Hi-C heatmap, information on both species (*S. aterrima* and *S. pratorum*) was obtained simultaneously (Table 4, Fig. 1b). Possible contaminants were detected using MMseqs. 2 v11^25^, which performed BLASTN-like searches based on the NCBI nucleotide (nt) and UniVec databases. The completeness of the genome was evaluated using BUSCO v3.0.2^26^ with insecta_odb10 dataset (n = 1,367 single-copy orthologues) and BUSCO v5.4.4^27^ with diptera_odb10 dataset (n = 3,285 single-copy orthologues). To calculate the mapping rate, we mapped ONT long reads and BGI short reads to the assembly using Minimap2. We then calculated the mapping rate using SAMtools v.1.10^28^ with the ‘flagstat’ parameter. Finally, the genomes of *S. aterrima* and *S. pratorum* were assembled into three chromosomes with sizes of 78.45 Mb and 71.56 Mb, the scaffold N50 lengths evaluated with insecta_odb10 dataset were 25.73 Mb and 23.53 Mb, while the GC content was 36.93% and 41.72%, respectively. (Table 5). The results evaluated with diptera_odb10 dataset in Table 6.

**Table 4:** The Chromosome length and sequencing coverage of *S. aterrima* (SateA) and *S. pratorum* (SateB).

Chromosome	Chromosome length (Mb)	Sequencing coverage (×)
SateA_Chr1	25.73	123.91
SateA_Chr2	23.35	124.04
SateA_Chr3	29.37	125.51
SateB_Chr1	23.53	38.45
SateB_Chr2	21.74	40.37
SateB_Chr3	26.29	39.60

**Table 5:** Genome assembly of *S. aterrima* (SateA) and *S. pratorum* (SateB) with insecta_odb10 dataset.

Assembly	Total length (Mb)	Number of scaffolds/contigs	scaffold/contigs N50 length (Mb)	Longest scaffold/contigs (Mb)	GC (%)	BUSCO (n = 1,367) (%)			
						C	D	F	M
SateA	78.454	3/60	25.732/2.195	29.372/7.074	36.93	98.5	1.3	0.2	1.3
SateB	71.559	3/57	23.533/2.953	26.286/5.125	41.71	97.8	0.9	0.5	1.7

Note: BUSCO, Benchmarking Universal Single-Copy Orthologs; C, complete BUSCOs; D, complete and duplicated BUSCOs; F, fragmented BUSCOs; M, missing BUSCOs.

**Table 6:** Genome assembly of *S. aterrima* (SateA) and *S. pratorum* (SateB) with diptera_odb10 dataset.

Assembly	Total length (Mb)	Number of scaffolds/	scaffold/contigs N50 length (Mb)	Longest scaffold/contigs (Mb)	GC (%)	BUSCO (n = 3,285) (%)			
		contigs				C	D	F	M
SateA	78.454	3/60	25.732/2.1951	29.374/7.074	39.93	93.3	1.3	1.2	5.5
SateB	71.559	3/57	23.533/2.953	26.286/5.125	41.71	91.9	0.9	1.2	6.9

Note: BUSCO, Benchmarking Universal Single-Copy Orthologs; C, complete BUSCOs; D, complete and duplicated BUSCOs; F, fragmented BUSCOs; M, missing BUSCOs.

### Genome annotation

Genomes are often annotated with repeat sequences, protein-coding genes, and non-coding RNA.

We used the software RepeatModeler v2.0.2a^29^ with an LTR discovery pipeline (-LTRStruct) to construct a repeat DNA library. Then the Dfam 3.3^30^ and RepBase-20181026^31^ databases were merged into a custom library, and finally the software RepeatMasker v4.1.2p1^32^ with the default commands was used to predict the repeat sequence according to the custom library. The genomes of *S. aterrima* and *S. pratorum* produced a total of 27,699 repeats (5.68 Mb) and 15,775 repeats (1.94 Mb), respectively, resulting in a repeat sequence ratio of 7.24% and 2.72%. The five most prevalent classes of repeat sequences were unknown (4.40% and 1.25%), LTR elements (1.13% and 0.62%), DNA elements (0.68% and 0.23%), Simple repeats (0.44% and 0.20%), and LINEs (0.30% and 0.19%). Statistical results are shown in Tables S1, S2.

The protein-coding genes were annotated by integrating the evidence of *ab initio*, transcriptome-based prediction, and homology-based annotations. The protein coding gene structures were predicted using MAKER v3.01.03 with the default commands^33^. For the predictions of *ab initio*, BRAKER v2.1.6^34^ and GeMoMa v1.8^35^ were used to integrate the transcriptomic and protein evidence and to integrate the predicted results of both as the input file for MAKER *ab initio* (ab. gff3). The transcriptome was aligned with the RNA-seq data to the genome by HISAT2 v2.2.0^36^ to generate BAM files. Augustus v3.3.4^37^ and GeneMark-ES/ET/EP 4.68_3.60_lic^38^ were automatically trained by BRAKER^39^, and integrate arthropod protein sequences (OrthoDB10 v1 database^40^) to improve the prediction accuracy. We used RNA-seq alignments produced from HISAT2 to perform genome-guided assembly by StringTie v2.1.6^41^. For the homology-based approach, GeMoMa with GeMoMa. c = 0.4 GeMoMa. p = 10 parameter was used to perform the annotation of protein-coding based on the annotation of genes of *Anopheles arabiensis* (GCF_016920715.1), *Bradysia coprophila* (GCF_014529535.1), *Culex quinquefasciatus* (GCF_015732765.1), *Drosophila melanogaster* (GCF_000001215.4), and *Hermetia illucens* (GCF_905115235.1) from GenBank. Finally, we predicted a total of 12,330 and 11,250 protein-coding genes in *S. aterrima* and *S. pratorum*, respectively. These genes had an average of 5.2/5.2 exons per gene, with an average exon length of 429.3/414.5 bp, and an average of 4.1/4.1 introns per gene, with an average intron length of 416.7/414.5 bp. Further, each gene contained 4.9/5.0 CDS, with an average CDS length of 344.4/342.8 bp. BUSCO completeness of the protein sequences was 97.7%/97.4% (n = 1,367), including 75.4%/74.4% single-copy, 22.3%/23.0% duplicated, 0.1%/0.4% fragmented, and 2.2%/2.2% missing BUSCOs, suggesting high-quality predictions.

Non-coding RNAs including transfer RNAs (tRNAs), microRNAs (miRNAs), ribosome RNAs (rRNAs), and small nuclear RNAs (snRNAs) were also identified. The rRNAs, snRNAs, and miRNAs were detected from the Rfam database (release 13.0)^42^ using Infernal v1.1.4^43^. The tRNAs were predicted using tRNAscan-SE v2.0.9^44^ with the script “EukHighConfidenceFilter”. The rRNAs and subunits were predicted using RNAmmer v1.2^45^. We identified a total of 273 and 273 noncoding RNA sequences were annotated for *S. aterrima* and *S. pratorum*. These included 35 and 34 microRNAs (miRNAs), 26 and 42 ribosomal RNAs (rRNAs), 26 and 20 small nucleolar RNAs (snRNAs), 137 and 123 tRNAs, and 45 and 50 other RNA sequences, respectively. The snRNAs identified included 15 and 9 spliceosomal RNAs (U1, U2, U4, U5, U6), 10 C/D box snoRNAs, and 1 HACA-box snoRNA in each species, respectively (Tables S3, S4).

Two strategies were used for the annotation of gene functions. We conducted the gene functional annotation search against the UniProtKB (SwissProt + TrEMBL)^46^ and the nonredundant protein sequence database (NR) using the sensitive mode of Diamond v2.0.11.149^47^ in sensitive mode with the parameters “–very-sensitive -e 1e-5”. We further employed eggNOG-mapper v2.1.5^48^ and InterProScan 5.53‐87.0^49^ to assign Gene Ontology (GO), Kyoto Encyclopedia of Genes and Genomes (KEGG) and Reactome pathway annotations and to identify protein domains. Four databases including protein families (Pfam)^50^, SMART^51^, Superfamily^52^, CDD^53^ were searched by InterProScan. The results predicted by the above tools were integrated to obtain the final prediction of gene functions. For *S. aterrima* and *S. pratorum*, a high percentage of annotated genes matched the UniProtKB database, with 11,740 (95.21%) and 10,835 (96.31%) genes respectively. The InterProScan database identified protein domains in 9,419/8,811 protein-coding genes, while 10,135/9,447 GO and 4,746/4,491 KEGG were identified by InterProScan and eggNOG-mapper. Furthermore, 7140/6695 genes were annotated as GO terms, 7673/7202 as KEGG ko terms, 2749/2614 as Enzyme Codes, 4746/4491 as KEGG pathways, 9419/8811 as Reactome pathways, and 10762/10047 as COG functional categories (Table 7).

**Table 7:** Genome annotation and functional annotation results of *S. aterrima* and *S. pratorum*.

Annotation	*S. aterrima*	*S. pratorum*
**Structure annotation**		
Number of protein-coding genes	12,330	11,250
Number of predicted protein sequences	16,990	15,391
BUSCO completeness (%)	97.7	97.4
Mean protein length (aa)	608.6	600.5
Mean gene length (bp)	3,670.6	3,664.8
Gene ratio	30.17%	27.48%
Number of exons per gene	5.2	5.2
Mean exon length (bp)	429.3	414.5
Exon ratio	18.39%	16.36%
Number of CDSs per gene	4.9	5.0
Mean CDS length (bp)	344.4	342.8
CDS ratio	14.06%	12.95%
Number of introns per gene	4.1	4.1
Mean intron length (bp)	416.7	419.0
Intron ratio	11.78%	11.12%
**Function annotation**		
Number of genes matching Uniprot records	11,740	10,835
Number of genes labelled as “Uncharacterized protein”	129	113
Number of genes labelled as “unknown function”	618	431
Number of genes with GO items from InterProScan annotations	7,140	6,695
Number of genes with MetaCyc items from InterProScan annotations	7,379	6,888
Number of genes with Reactome items from InterProScan annotations	9,419	8,811
Number of genes with eggNOG annotations	11,360	10,562
Number of genes with GO items from eggNOG annotations	9,006	8,430
Number of genes with Enzyme Codes (EC) from eggNOG annotations	2,749	2,614
Number of genes with KEGG ko terms from eggNOG annotations	7,673	7,202
Number of genes with KEGG pathway terms from eggNOG annotations	4,746	4,491
Number of genes with COG Functional Categories from eggNOG annotations	10,762	10,047
Number of genes with GO items (combining InterProScan and eggNOG results)	10,135	9,447
Number of genes with KEGG pathways items (combining InterProScan and eggNOG results)	4,746	4,491

## Data Records

All raw sequencing data and the genome assembly of *S. aterrima* and *S. pratorum* underlying this article are available at the NCBI and can be accessed with Bioproject ID PRJNA809421. The Nanopore, Illumina, Hi-C, and transcriptome data can be found under identifcation numbers SRR23797681-SRR23797685^54–58^. The assembled genome has been deposited in the NCBI assembly with the accession number GCA_033063855.1^59^ and GCA_033064975.1^60^. All annotations data for repeated sequences, gene structure, and functional prediction are available for download through Figshare^61^ (10.6084/m9.figshare.22762118).

## Technical Validation

Two genome assembly methods, NextDenovo, and 3D-DNA, were executed and subsequently compared (Table 3). The NextDenovo assembly genome was slightly larger than predicted at 151.48 Mb and contained 78 primary contigs, with an N50 contig length of 3.39 Mb, the longest contig of 9.63 Mb, and 98.40% of BUSCO genes. The average GC content was 39.16%. After haplotig purging and genome polishing, the genome length was 151.31 Mb, contained 78 primary contigs with an N50 contig length of 3.38 Mb, the longest contig of 9.62 Mb, and 99.10% of BUSCO genes. The 3D-DNA assembly showed a remarkable improvement, yielding 89 scaffolds with a scaffold N50 length of 25.73 Mb, including the longest scaffold of 29.79 Mb. Additionally, the BUSCO completeness percentage achieved in the 3D-DNA version was 99.2%, with negligible levels of fragmentation and missing genes at 0.00% and 0.80% respectively. After utilizing Hi-C long-range scaffolding to enhance our assembly, we securely anchored the assembled scaffolds into six chromosomes that can be categorized into two species; *S. aterrima* (SateA) and *S. pratorum* (SateB) (Fig. 1b). Subsequently, we imported the 3D-DNA assembly to obtain the final results of the chromosome assembly.

The final genome assembly showed a BUSCO completeness of 98.5% and 97.8% (n = 1,367), and duplicated BUSCOs were 1.3% and 0.9%, respectively. Additionally, the high mapping ratios of both BGI and ONT data were 93.38% and 92.35%, respectively. These indicators collectively demonstrate that the assembly has achieved a remarkable degree of continuity and integrity (Table 5).

### Supplementary information

Supplementary tables

## Data Availability

The versions of software used were included in the methods section. No specific script was utilized for this work. All commands and pipelines employed in data processing were executed in accordance with the manual and protocols of the corresponding bioinformatic software.
